# Personal

**Published:** 1893-06

**Authors:** 


### Personal.

DR. M. A. CROCKETT has removed his office to 452 Franklin street.

DR. H. CORWIN JONES has located his office at 255 Eighteenth street.

DR. CHARLES CARY has returned from his European trip much improved in health.

DR. COAKLEY has been elected a City councilman to fill the vacancy caused by the death of George W. Hayward.

DR. F. S. CREGO expects to be installed in his beautiful new office, corner Delaware avenue and Virginia streets, before the close of the month.

DR. GEORGE E. FELL has resigned his position as Professor of Physiology and Microscopy in the Medical Department of Niagara University.

DR. ERNEST WENDE has removed his office temporarily to 467 Virginia street, awaiting the completion of his new home on Delaware avenue.

DR. EVANGELINE CARROLL, of Ashland avenue, has left the city to assume the position of senior physician in the Woman's Hospital in Detroit.

DR. FREDERICK L. VAN SICKLE has removed from 729 Prospect avenue to 393 Hampshire street, corner Eighteenth. Hours: 8 to 10 A. M.; 1 to 3, and 7 P. M.

Dr. Louis A. Bull, whose practice is limited to diseases of the nose, throat, and chest, has removed his office to Nos. 32 and 34 Market Arcade, 619 Main street, Buffalo, N. Y.

---

Dr. Brooks H. Wells, editor of the *American Journal of Obstetrics*, has recently been appointed Adjunct-Professor of Gynecology at the New York Polyclinic. The Polyclinic has done itself great honor.

---

Dr. A. L. Benedict, who is making a specialty of diseases of the digestive organs—stomach, intestine, and liver—is now located at 174 Franklin street, Hours: 8 to 9 a. m., 2 to 3, and 6.30 to 7.30 p. m.; Sundays, 12 m.

---

Dr. Irving M. Snow, whose practice is confined to diseases of children, has removed his office and residence from 371 Porter avenue to 476 Franklin street, near Allen. Office hours: 8 to 9.30 a. m., 2 to 3, and 7 p. m.

---

Dr. George Ben Johnston, of Richmond, Va., has been unanimously chosen by the Trustees of the University of Virginia, as Professor of Surgery of the Medical Department vice J. S. Dorsay Cullen, M. D., deceased. The Richmond faculty is to be congratulated upon the accession of so competent a colleague.

---

Dr. Walter P. Manton, of Detroit, was elected an honorary member of the Kalamazoo Academy of Medicine, at its annual session in January last, at which time he read a paper on the Diagnosis of Abdominal Tumors, and exhibited several specimens illustrating the points made in his instructive communication.

---

Professional Aristocrats.—Our friend, Dr. W. H. Wathen, returned from the East, yesterday morning, looking fresh and cheerful, and no wonder, for he had been attending the meeting of the National Association of Gynecologists in Philadelphia. This society embraces the aristocracy of the profession. Dr. Wathen says that at the recent meeting there were ten applicants for membership, and seven were rejected.—*Louisville Commercial.*

